# Investigation the effect of jujube seed capsule on sleep quality of postmenopausal women: A double-blind randomized clinical trial

**DOI:** 10.37796/2211-8039.1038

**Published:** 2020-12-01

**Authors:** Razieh Mahmoudi, Somayeh Ansari, Mohammad Hosein Haghighizadeh, Nader Shakiba Maram, Simin Montazeri

**Affiliations:** a Department of Midwifery, Faculty of Nursing and Midwifery, Ahvaz Jundishapur University of Medical Sciences, Ahvaz, Iran; b Menopause Andropause Research Center, Department of Midwifery, Faculty of Nursing and Midwifery, Ahvaz Jundishapur University of Medical Sciences, Ahvaz, Iran; c Department of Biostatistics, School of Health, Ahvaz Jundishapur University of Medical Sciences, Ahvaz, Iran; d Ahvaz Jundishapur Nanotechnology Research Center, Ahvaz, Iran; e Department of Midwifery, Reproductive Health Promotion Research Center, Ahvaz Jundishapur University of Medical Sciences, Ahvaz, Iran

**Keywords:** Sleep quality, Postmenopausal women, Jujube, Iran

## Abstract

**Bakground and objective:**

Sleep disorder is among the most common problems in the life of postmenopausal women. Because of the complications of chemical drugs, many women prefer to use herbal supplements for relieving sleep problems. So, the main objective of this study was to determine the effect of the jujube seed capsule on sleep quality in postmenopausal women.

**Materials and methods:**

This study was a double-blind clinical trial conducted on 106 postmenopausal women in Khuzestan province, southwest of Iran. All participants were selected by a simple non-probability sampling method. Data were collected through a demographic data form and the Pittsburgh sleeps quality index (PSQI). Individuals were randomly divided into intervention (*n* = 53) and control (*n* = 53) groups. The intervention group received 250 mg oral jujube seed capsule and the control group received a placebo capsule twice a day for 21 days. After the treatment, the PSQI was completed in both intervention and control groups. Data were analyzed using the independent t-test and the Chi-square test using SPSS software version 24, and *p*-value < 0.05 was considered as the significance level.

**Results:**

The results revealed that after treatment, the mean scores of sleep quality decreased in the intervention and control group. Although this difference was statistically significant in both intervention and control groups (*p*-value < 0.05), more reduction observed in the intervention group (*p*-value < 0.001).

**Conclusion:**

Consumption of the jujube seed capsule had a positive impact on improving the sleep quality of postmenopausal women and could be recommended as a useful herbal medication.

## 1. Introduction

Menopause is a natural part of the life cycle of women that occurs in the middle or late middle ages [1]. World Health Organization (WHO) considered menopause as the real stop of menstruation for 12 months due to loss of ovarian follicles activity [2]. It estimated that the population of 467 million postmenopausal women would reach 1,200 million ones by 2030 [3]. Postmenopausal age changes usually occur in the age range of 45-55 years [4]. The studies on postmenopausal age in different Iranian cities have reported a wide range of natural postmenopause from 46.01 to 53.4 years [5]. The main reason for the emergence of postmenopausal symptoms is reduced ovarian estrogen levels. The level of estrogen in postmenopausal women accounts for about 10% of young women [6]. In menopause, due to lower levels of the hormone in the body, women undergo many changes such as hot flash, night sweats, palpitations, headaches, dizziness, fatigue, and irritability. One of the most common problems among these women is sleep disorder [7]. These symptoms appear as partial discomfort to severe and disabling symptoms in different individuals [8]. Sleep is a repetitive state and rest time for the body and mind that causes physical and mental renewal, which referred to as the most basic human needs [9]. Human beings devote about one-third of their lives to sleep [8]. Although the need for sleep in people is completely different and varies from childhood to old age, all age groups need sleep and rest during the night such that an adult need at least 8 hours of sleep per day within 4-6 cycles of 90 minutes [10]. About one-third of adults in the world suffer from the sleep disorder. This problem is exacerbated by increasing the age and addition of chronic diseases and increases up to about 69%. In this regard, the prevalence of sleep disorder has been reported as about 65% among postmenopausal women, and it is one of the main reasons for visiting medical centers by postmenopausal women [11]. Sleep disorders have negative impacts on quality of life and cause a reduction in daily functioning in terms of physical, psychological, and social aspects [12]. Besides, a sleep disorder can reduce cognitive performance and cause an error in works, obesity, change in body metabolic mechanisms, and even death due to deprivation [8]. Different studies have indicated that the quality of sleep in postmenopausal women is not optimal. For example, Azhari et al. showed that most postmenopausal women (73%) had bad sleep quality [13]. The first line of treatment for treating sleep disorders in postmenopausal women is the administration of hormones. This treatment has several complications such as breast tenderness, nausea, and headache for postmenopausal women, which makes them unwilling to use this approach [14]. In addition to hormonal medications, sedative and hypnotic drugs are also considered as an option. However, these drugs also cause various complications such as drowsiness, exacerbation of sleep disorders, dry mouth, and constipation [15]. In addition to hormone and chemical drugs, various complementary therapies such as therapeutic touch, aromatherapy, relaxation, music therapy, yoga, acupuncture, and medicinal plants have been provided for the treatment of sleep problems in postmenopausal women [16]. Today, the WHO and many researchers have paid particular consideration to the side effects of chemical drugs, the prohibition of using these drugs in some individuals, and the tendency to use herbal medicines. The jujube plant with the scientific name of the Chinese date and Jujube tree is from the Rhamnaceae family [17]. Jujube is widely found in northern China, and its seeds are used for the treatment of insomnia, wounds, and injuries, stimulating the immune system, anticancer properties, the nervous system protection, and anti-inflammatory properties [17,18]. The jujube has compounds like phenol, flavonoid, and saponin. These compounds cause hypnotic effects through influencing gamma-aminobutyric acid (GABA) system neural transmitters [19]. Also, these compounds impose their impacts on affecting serotonin and serotonergic system that play a significant role in the sleep cycle [20]. Palmieri et al. showed that plant compounds such as jujube, valerian, and hops improved sleep parameters [21]. Moreover, Fang et al. indicated that jujube and sage aquatic extract could shorten the duration of sleep latency in the mouse samples and increase the duration of sleep [22]. San et al. showed that the administration of oral jujube extracts with an intraperitoneal injection of sodium pentobarbital significantly increased the sleep time in mice [19]. In the Chinese ancient medicine, jujube was consumed as a tea against insomnia. Definitely, the jujube has established to rise the pentobarbital-induced sleep time and diminish free movement on mice [23]. Therefore, considering increasing population of postmenopausal women and their sleep problems, fewer side effects involved in using herbal medicines compared to the chemical drugs, and lack of any study on the effect of jujube on the sleep quality in postmenopausal women, the current study aims at determining the impact of jujube seed capsule on postmenopausal women's sleep quality.

## 2. Patients and methods

### 2.1. Ethical consideration

This study was permitted by the Ethics Committee of Ahvaz Jundishapur University of Medical Sciences, Ahvaz, Iran under the Declaration of Helsinki (Ref No: IR.AJUMS.REC.1396.1056). Also, the protocol was registered at the Iranian Clinical Trials Registration (IRCT20180307038992N1) and the central office of the University of Medicinal Plants and Natural Plant Research Center under number A182430302FP. After explaining the aims of this research to all participants, the written informed consent form was taken from them.

### 2.2. Study design and data collection

This double-blind clinical trial conducted on 106 postmenopausal women referred to health centers of Haftkal town in Khuzestan province, southwest of Iran. Data were collected through a demographic data form and the Pittsburgh sleeps quality index (PSQI). Demographic data form included age, job, level of education, residence location, marital status, economic situation, duration of postmenopause, and the number of children. The PSQI developed by Buysse et al., which assesses sleep quality over the last month [24]. The PSQI comprises of nineteen self-report objects which combined to form seven component scores. The seven components include daytime dysfunction, sleep latency, subjective sleep quality, sleep duration, habitual sleep efficiency, sleep disturbances, and use of sleep medication. Each component score has a range of 0 to 3, and a score >1 designates problems in that feature of sleep quality. The total score of sleep quality ranges from 0 to 21 with a higher score demonstrating lesser quality of sleep, whereas a cutoff score of ≤5 specifies good sleep quality [25,26]. The PSQI had received extensive support to confirm its good psychometric properties and high correlation with actual sleep log data [27]. Good reliability of the PSQI was also demonstrated among 793 Chinese adults, with a Cronbach's alpha =0.84 and a 2-week test-retest reliability of 0.81 [28].

### 2.3. Production of jujube seed capsule

The jujube was prepared from local spice stores in Ahvaz city and authenticated at the Herbarium of the Department of Pharmacognosy, Faculty of Pharmacy, Ahvaz Jundishapur University of Medical Sciences, Ahvaz, Iran. Jujube seed capsule was manufactured in the laboratory of the Pharmacy faculty of Ahvaz Jundishapur University of Medical Sciences. After separating the jujube seed and washing, the pharmacist placed it at ambient temperature to dry and then powdered it with a grinder. Then, the resulting powder was formulated in zero size capsules using the device. The control group took a placebo capsule that was completely similar to the 250 mg jujube seed capsule in terms of shape and size. The placebo capsule contained Starch, Avicel, and Methylcellulose. Jujube seed and placebo capsules were separately placed in A and B packets by a pharmacist. Both the researcher and the subjects were unaware of the content of packets.

### 2.4. Participants and sampling method

Informed written consent was obtained from each participant before data collection. In this study, 106 postmenopausal women referred to health centers of Haftkal town were selected by simple non-probability sampling. Then the samples were randomly assigned to jujube seed capsule (*n* = 53) and placebo capsule (*n* = 53) groups with four block designs. A randomized list is provided by a statistician. The drugs used in this study are placed in sealed envelopes pocket according to the corresponding codes by a person out of the study and then assigned to each patient enrolled in the study. The medications were identical in terms of appearance such as packaging and color and thus, the researcher and patient were not aware of the type of drug. According to the statistical formula, the sample size was determined using 80% power, α = 0.05, *Z_β-1_* = 1829, *Z_1-_*_α_*_/2_* = 1.95, *S_1_* = 0.46, *S_2_* = 0.49, *X_1_* = 0.62, *X_2_* = 0.3, and considering the probability of a 10% drop out according to Taavoni et al. protocol [11].

$$n=\frac{{\left(Z_{1-\alpha /2}+Z_{1-\beta }\right)}^{2}\left(S_{1}^{2}+S_{2}^{2}\right)}{{\left(X_{1}-X_{2}\right)}^{2}}$$

### 2.5. Inclusion and exclusion criteria

Inclusion criteria included age range of 45-65 years, passing at least 1 year after the last menstruation, and earning sleep quality score of ≥5 based on PSQI. Exclusion criteria included taking alcohol and smoking, taking other hormonal and herbal medicines, taking drugs affecting sleep (diuretics, tranquilizers, and antidepressants), occurrence of any physical and mental illness during the study causing sleep disorders (dyspnea and thyroid dysfunction), and emergence of possible and significant changes in sleep conditions unpredictably (travel, displacement, and severe stress).

### 2.6. Intervention protocol

**Fig. 1:**
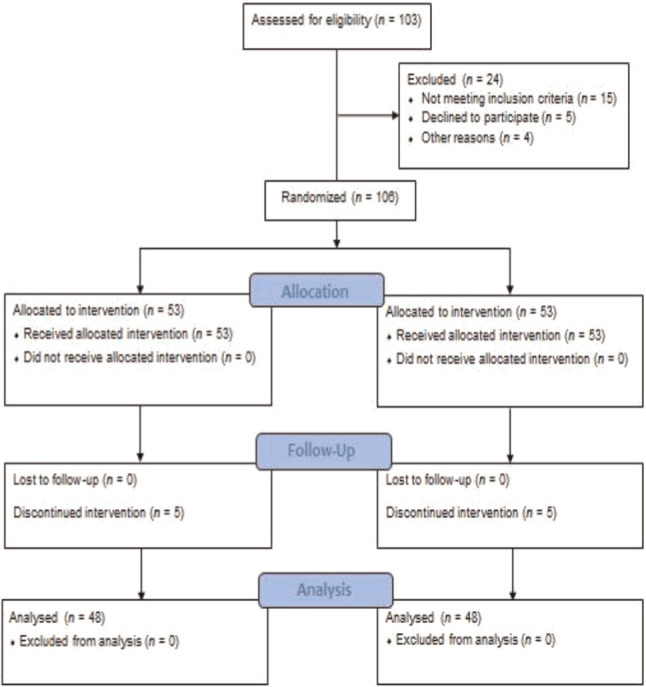
Flow chart of the study.

Each of the capsules used was randomly placed in packed envelopes according to a randomized list by an individual who was excluded from the study and delivered to each patient who met the inclusion criteria. The drugs were quite similar in appearances, such as packaging and color, so the researcher and patient were completely unaware of the type of drug. The intervention in the jujube seed capsule group consisted of oral administration of 250 mg jujube seed capsule twice a day and the duration of using it according to the study was two tablets daily at bedtime for 21 days [26]. Also, the control group received a placebo capsule that was completely similar to the 250 mg jujube seed capsule in terms of shape and size. To supervise the consumption of capsules, the researcher controlled the jujube consumption by the research units through a phone call, and the samples were asked to immediately inform the researcher in case of any problem with consuming the capsules, need for consumption of other drugs, or tendency to exit the study. Both groups were followed up regularly by the researcher. Finally, after one month of treatment and referral to the treatment center, the PSQI was completed between the two groups again. In total, 96 postmenopausal women took part in the study and 10 women were excluded due to digestive problems and reluctance to continue treatment (Fig. 1).

### 2.7. Analysis of data

All statistical analyses were done using SPSS™ software version 24.0 (IBM Corporation, Armonk, NY, USA). The Kolmogorov–Smirnov test was used for testing the normal distribution. An independent *t*-test was used to compare quantitative variables between the two groups. Also, the *chi*-square test was used to compare the qualitative variables between the two groups. The *p*-value <0.05 was considered as a significant level.

## 3. Results

In current research, of 106 participants, a sum of 96 postmenopausal women had the inclusion criteria. The results obtained from this study showed that no significant variance is observed between the two groups in job, level of education, residence location, marital status, and economic situation using the *chi*-squared test, and mean age, duration of postmenopause, and the number of children by using the independent *t*-test (*p* > 0.05) (Table 1).

Table 2 showed that the total mean score of sleep quality decreased after intervention in the jujube seed capsule compared to before the intervention (*p* < 0.001). Besides, based on the pairwise *t*-test, there was a statistically significant difference between the total mean score of sleep quality in the intervention group compared to control group. However, changes in the total mean score of sleep quality were higher in the intervention group than the control group. According to independent *t*-test results, there was no statistically significant difference between the total mean score of sleep quality before intervention in both groups (*p* = 0.58). However, the total mean score of sleep quality showed a significant difference after intervention in the case and control groups (*p* < 0.001).

## 4. Discussion

The current research was conducted to determine the impact of the jujube seed capsule on the sleep quality of postmenopausal women. Findings of current research indicated that there was a statistically significant difference in the total means score of PSQI between intervention and control groups after the intervention. Therefore, it can be stated that the jujube seed capsule can be effective as a plant medicine for improvement and enhancement of sleep quality in postmenopausal women. The findings of the current study were consistent with the study results of Palmieri et al., who showed that plant compounds of jujube, valerian, and hops could improve sleep parameters in comparison to placebo [21]. In the study of Scholey et al., aimed at determining the effect of Lactium and Zizyphus Complex on sleep quality, the results showed that the administration of LZComplex3 in a short time may have masked a potential beneficial effect on sleep quality [29]. So, this finding was consistent with the current research. One of the important aspects of the current study was its innovation in its field. Despite extensive search in scientific texts, no other studies were found on assessing the impact of jujube on sleep quality of postmenopausal women. Additionally, no other reports were found with results contradictory to the findings of the current research. Although there are few human articles about the effect of jujube on sleep quality, in other animal studies conducted on mice, the effect of jujube on sleep disorders was visible. In laboratory studies, relaxing impacts of jujube on sleep and its impact on sleep disorders can be observed. The result of the study of San et al. showed that oral administration of the 50% ethanolic extract from Ziziphus mauritiana seeds at the dose of 200 mg/kg significantly increased the sleeping time in mice intraperitoneally administered with sodium pento-barbital (50 mg/kg body weight) [19]. Therefore, the results of their study in terms of improving sleep quality after oral administration are consistent with the present study. In a study by Kao et al. in China, it was reported that the sleeping effect of jujube on mice is because of its impact on the serotonergic system, and jujube is a new source of control and adjustment of the sleep mechanism [20]. A study by Farag and Mills in the US aimed at determining the effect of traditional herbal supplements on insomnia showed a significant decrease in delayed sleep initiation in traditional herbal supplement users [30]. These results were confirmed by current research which showed the consumption of the herbal medicine can improve the sleep quality. The phenolics, flavonoids, and triterpenic saponins are among the active components of jujube [23,31,32]. The triterpenic saponins may be useful for lightening of sleep disorders through the modulation of the monoaminergic system [23,31,33,34]. In a clinical trial that evaluate the usefulness of sour date (*Zizyphus jujube var. spinosa*) in the popular Chinese herbal form of suanzaorentang on sleep quality of 60 participants with insomnia, all scores of sleep quality meaningfully improved during the treatment period [35,36,37,38]. It seems that combination of jujube with other drugs or herbs can lead to the production of compounds that are effective in improving of sleep quality [39,40].

The limitations of current research are the lack of investigation of sleep disorder reason and the impact of confounding factors on sleep quality. Since some participants were illiterate and the questionnaire was completed by the researcher, the accuracy of information by the samples is not reliable. Also, although sleep quality is a qualitative issue, it was measured with a quantitative tool. Despite the novelty of the present study, there was a limitation in data interpretation due to not finding any similar study.

## 5. Conclusions

The results show that the medicinal jujube seed capsule improved the sleep quality of postmenopausal women. Thus, it could be recommended as a safe plant with fewer complications compared to chemical and hormone drugs for the treatment of sleep disorders. Also, during the period of consuming jujube seed capsule in this study, except for a few cases of gastrointestinal disorders and in similar studies with this herbal medicine, consumers have reported no specific side effects. Therefore, considering that about one-third of adults in the world suffer from sleep disorders, the high prevalence of sleep disorders in postmenopausal women, and the lower cost of treatment by jujube seed capsule compared to other treatments, it is suggested implementing this treatment as a useful method in improving sleep quality in postmenopausal women.

## Supplementary materials



## Figures and Tables

**Table 1:** Distribution and mean value of samples based on demographic information of participants in the intervention group and control group.

Variable		Group		
		Intervention group	Control group	*p*-value
Age (year), Mean ± SD		58.45 ± 5.60	57.06 ± 6.54	0.26
Duration of postmenopause (year), Mean ± SD		11.66 ± 7.10	9.8 ± 7.009	0.76
Number of children, Mean ± SD		5.43 ± 2.74	5.31 ± 2.95	0.83
Education level, n (%)	> Diploma	47 (97.9)	43 (89.6)	0.47
	Diploma	-	3 (6.3)	
	< Diploma	1 (2.1)	2 (4.2)	
Residence location, n (%)	City	26 (54.2)	29 (60.4)	0.53
	Village	22 (45.8)	19 (39.6)	
Marital status, n (%)	Single	2 (4.2)	2 (4.2)	0.48
	Married	37 (77.1)	41 (85.4)	
	Widow	9 (18.8)	5 (10.4)	
Economic situation, n (%)	Good	1 (2.1)	4 (8.3)	0.37
	Average	34 (70.8)	33 (68.8)	
	Poor	13 (27.1)	11 (22.9)	
Job, n (%)	Housewife	47 (97.9)	43 (89.6)	0.92
	Employed	1 (2.1)	5 (10.4)	

**Table 2:** Comparison of the total score of sleep in postmenopausal women in two groups before and after the intervention.

Overall sleep quality	Group		
	Intervention group	Control group	*p*-value
	Mean ± SD		
Before intervention	10.47 ± 2.97	10.16 ± 2.65	0.58
After intervention	6.22 ± 2.52	9.25 ± 3.27	<0.001
*p*-value	<0.001	<0.001	
