# Supplementary material for: Investigation the effect of jujube seed capsule on sleep quality of postmenopausal women: A double-blind randomized clinical trial
Source: Biomedicine (Taipei). 2020 Dec 1;10(4):42–8. doi: 10.37796/2211-8039.1038 (PMC7735973; doi:10.37796/2211-8039.1038)
Supplement: Supplementary file 1 [file bmed-10-04-042-s001.docx]

**Abstract**

**Bakground and objective:** Sleep disorder is among the most common problems in the life of postmenopausal women. Because of the complications of chemical drugs, many women prefer to use herbal supplements for relieving sleep problems. So, the main objective of this study was to determine the effect of the jujube capsule on sleep quality in postmenopausal women.

**Materials and methods:** This study was a double-blind clinical trial conducted on 106 postmenopausal women in Khuzestan province, southwest of Iran. All participants were selected by a simple non-probability sampling method. Data were collected through a demographic data form and the Pittsburgh sleeps quality index (PSQI). Individuals were randomly divided into intervention (*n* = 53) and control (*n* = 53) groups. The intervention group received 250 mg oral jujube capsule and the control group received a placebo capsule twice a day for 21 days. After the treatment, the PSQI was completed in both intervention and control groups. Data were analyzed using the independent t-test and the Chi-square test using SPSS software version 24, and *p*-value < 0.05 was considered as the significance level.

**Results:** The results revealed that after treatment, the mean scores of sleep quality decreased in the intervention and control group. Although this difference was statistically significant in both intervention and control groups (*p*-value < 0.05), more reduction observed in the intervention group (*p*-value < 0.001).

**Conclusion:** Consumption of the jujube capsule had a positive impact on improving the sleep quality of postmenopausal women and could be recommended as a useful herbal medication.

**Keywords**: Sleep quality, Postmenopausal women, Jujube, Iran
