# Supplementary material for: Investigation the effect of jujube seed capsule on sleep quality of postmenopausal women: A double-blind randomized clinical trial
Source: Biomedicine (Taipei). 2020 Dec 1;10(4):42–8. doi: 10.37796/2211-8039.1038 (PMC7735973; doi:10.37796/2211-8039.1038)
Supplement: Supplementary file 2 [file bmed-10-04-042-s002.pdf]

BioMedicine

CONFLICTS OF INTEREST STATEMENT

Manuscript title: **Investigation the effect of jujube capsule on sleep quality of postmenopausal women: A double-blind randomized clinical trial**

The authors whose names are listed immediately below certify that they have NO affiliations with or involvement in any organization or entity with any financial interest (such honoraria; educational grants; participation in speakers' bureaus; membership, employment, consultancies, stock ownership, or other equity interest; and expert testimony or patent-licensing arrangements), or non-financial interest (such as personal or professional relationships, affiliations, knowledge or beliefs) in the subject matter or materials discussed in this manuscript.

Author names: **Simin Montazeri**

The authors whose names are listed immediately below report the following details of affiliation or involvement in an organization or entity with a financial or non-financial interest in the subject matter or materials discussed in this manuscript. Please specify the nature of the conflict on a separate sheet of paper if the space below is inadequate.

Author names: Razieh Mahmoudi, Somayeh Ansari, Mohammad Hosein Haghighizadeh, Nader Shakiba Maram, Simin Montazeri

This statement is signed by all the authors to indicate agreement that the above information is true and correct (a photocopy of this form may be used if there are more than 10 authors):

Author's name (typed)	Author's signature	Date
1. Razieh Mahmoudi		22.4.2020
2. Somayeh Ansari		22.4.2020
3. Mohammad Hosein Haghighizadeh		22.4.2020
4. Nader Shakiba Maram		22.4.2020
5. Simin Montazeri		22.4.2020
