# Supplementary material for: Investigation the effect of jujube seed capsule on sleep quality of postmenopausal women: A double-blind randomized clinical trial
Source: Biomedicine (Taipei). 2020 Dec 1;10(4):42–8. doi: 10.37796/2211-8039.1038 (PMC7735973; doi:10.37796/2211-8039.1038)
Supplement: Supplementary file 3 [file bmed-10-04-042-s003.pdf]

BioMedicine

AUTHORSHIP STATEMENT

Article Investigation the effect of jujube capsule on sleep quality of postmenopausal women: A double-blind randomized clinical trial

All persons who meet authorship criteria are listed as authors, and all authors certify that theyhave participated sufficiently in the work to take public responsibility for the content, including participation in the concept, design, analysis, writing, or revision of the manuscript. Furthermore, each author certifies that this material or similar material has not been and will not be submitted to or published in any other publication before its appearance in BioMedicine.

Authorship contributions

Please indicate the specific contributions made by each author (list the authors' initials followed by their surnames, e.g., Y.L. Chang). The name of each author must appear at least once in each of the three categories below.

Category 1

Conception and design of study: R. Mahmoudi, S. Ansari, M.H. Haghighizadeh, ____;____;

acquisition of data: N. Shakiba Maram, S. Montazeri, ____, ____;____;

analysis and/or interpretation of data: N. Shakiba Maram, S. Montazeri, ____, ____.____;

Category 2

Drafting the manuscript: R. Mahmoudi, S. Ansari, N. Shakiba Maram, S. Montazeri;

revising the manuscript critically for important intellectual content: R. Mahmoudi, S. Ansari, N. Shakiba Maram, S. Montazeri.

Category 3

Approval of the version of the manuscript to be published (the names of all authors must be listed):

R. Mahmoudi, S. Ansari, M.H. Haghighizadeh, N. Shakiba Maram, S. Montazeri, ____, ____, ____,

Acknowledgments

All persons who have made substantial contributions to the work reported in the manuscript (e.g., technical help, writing and editing assistance, general support), but who do not meet the criteria for authorship, are named in the Acknowledgments and have given us their written permission to be named. If we have not included an Acknowledgments, then that indicates that we have not received substantial contributions from non-authors.

This statement is signed by all the authors (a photocopy of this form may be used if there are more than 10 authors):

Author's name (typed) Author's signature Date

Simin Montazeri — Digitally signed by Simin Montazeri Date: 2020.04.22 13:32:11 +04'30'	Razieh Mahmoudi
Somayeh Ansari	Mohammad Hosein Haghighizadeh

Nader Shakiba Maram

Corresponding author declaration

I [ ] , the corresponding author of this manuscript, certify that the contributors' and conflicts of interest statements included in this paper are correct and have been approved by all co-authors
