# Supplementary material for: Investigation the effect of jujube seed capsule on sleep quality of postmenopausal women: A double-blind randomized clinical trial
Source: Biomedicine (Taipei). 2020 Dec 1;10(4):42–8. doi: 10.37796/2211-8039.1038 (PMC7735973; doi:10.37796/2211-8039.1038)
Supplement: Supplementary file 4 [file bmed-10-04-042-s004.docx]

Investigation the effect of jujube capsule on sleep quality of postmenopausal women: A double-blind randomized clinical trial

Running title: Effect of jujube on sleep of postmenopausal women

Razieh Mahmoudi^1^, Somayeh Ansari^2^, Mohammad Hosein Haghighizadeh^3^, Nader Shakiba Maram^4^, Simin Montazeri^5*^

^1^Department of Midwifery, Faculty of Nursing and Midwifery, Ahvaz JundiShapur University of Medical Sciences, Ahvaz, Iran

^2^Menopause Andropause Research Center, Department of Midwifery, Faculty of Nursing and Midwifery, Ahvaz Jundishapur University of Medical Sciences, Ahvaz, Iran

^3^Department Biostatistics, School of Health, Ahvaz Jundishapur University of Medical Sciences, Ahvaz, Iran

^4^ Ahvaz Jundishapur Nanotechnology Research Center, Ahvaz, Iran

^5^Department of Midwifery, Reproductive Health Promotion Research Center, Ahvaz Jundishapur University of Medical Sciences, Ahvaz, Iran

^*^Corresponding Author: Simin Montazeri

Address: Department of Midwifery, Reproductive Health Promotion Research Center, Ahvaz Jundishapur University of Medical Sciences, Golestan Blv. Ahvaz, Iran.

Email: siminmontazeri12@gmail.com

Postal Code: 61357-15794

P.O. Box: 159

Tel: +989386343150

Fax: +986133738331
